# HiFi-Assembled Mitogenomes of Four Pygmy Grasshoppers Reveal Mito–Nuclear Discordance in *Zhengitettix transpicula* and Lineage-Specific Mitochondrial Intergenic Length Variation

**DOI:** 10.3390/life16061015

**Published:** 2026-06-17

**Authors:** Rongjiao Zhang, Taihang Xu, Delong Guan, Weian Deng

**Affiliations:** 1 Guangxi Key Laboratory of Sericulture Ecology and Applied Intelligent Technology, Guangxi Collaborative Innovation Center of Modern Sericulture and Silk, Guangxi Colleges Universities Key Laboratory of Exploitation and Utilization of Microbial and Botanical Resources, School of Chemistry and Bioengineering, Hechi University, Hechi 546300, China; 06075@hcnu.edu.cn (R.Z.); 2021101786@hcnu.edu.cn (T.X.); 202366006@hcnu.edu.cn (D.G.); 2 Key Laboratory of Ecology of Rare and Endangered Species and Environmental Protection, Guangxi Normal University, Ministry of Education, Guilin 541006, China

**Keywords:** Tetrigidae, pygmy grasshoppers, mitochondrial genome, HiFi sequencing, mito–nuclear discordance, phylogeny, non-coding region, *Zhengitettix*

## Abstract

Mitochondrial genomes are widely used in insect taxonomy and phylogenetics, but their signals may conflict with morphology and nuclear genomic evidence because the mitochondrial genome represents a single maternally inherited locus. Here, we assembled complete mitochondrial genomes of four pygmy grasshoppers, *Zhengitettix transpicula*, *Formosatettix* sp., *Gibbotettix parvipulvillus*, and *Bolivaritettix* sp., using PacBio HiFi reads. The four mitogenomes ranged from 15,152 to 17,976 bp and contained the typical 37 mitochondrial genes. Mitochondrial phylogenies inferred by maximum likelihood and Bayesian methods were topologically identical and recovered several well-supported tetrigid relationships, including a close relationship between *Formosatettix* sp. and *Bolivaritettix* sp. However, *Z. transpicula* was unexpectedly placed near *Macromotettixoides* rather than close to other *Zhengitettix* representatives. In contrast, a morphology-based tree recovered *Z. transpicula* with *Z. triangularis*, and comparison with a published nuclear single-copy ortholog tree based on 1962 loci supported a non-mitochondrial placement of *Zhengitettix* inconsistent with the anomalous mitochondrial position of *Z. transpicula*. Independent assembly from the original HiFi reads, read-depth inspection, protein-coding gene checks, and nuclear-genome screening for NUMT-like sequences supported the authenticity of the assembled *Z. transpicula* mitogenome. These results document mito–nuclear and cyto-morphological discordance in Tetrigidae and highlight the need for integrative interpretation of mitochondrial phylogenies in taxonomically complex insect groups.

## 1. Introduction

Mitochondrial genomes have become one of the most frequently used genomic resources in insect systematics, taxonomy, and evolutionary biology. Owing to its high copy number, relatively conserved gene content, maternal inheritance, and rapid evolutionary rate, the mitochondrial genome has been widely applied in species identification, DNA barcoding, population genetics, and phylogenetic reconstruction [1,2,3]. In insects, mitogenomic data have been especially useful for groups in which morphological characters are limited, convergent, or difficult to interpret [4,5,6]. The rapid development of high-throughput sequencing has further accelerated the generation of complete mitochondrial genomes, making mitogenomics an increasingly accessible tool for linking classical taxonomy with evolutionary biology [3,5].

Despite these advantages, mitochondrial genomes represent only a single, maternally inherited genetic locus. Consequently, mitochondrial phylogenies may not always reflect the species tree, particularly in recently diverged lineages or groups affected by hybridization, introgression, incomplete lineage sorting, sex-biased dispersal, or mitochondrial capture [7,8,9]. Such mito-nuclear discordance has been documented in diverse animal groups and has important implications for taxonomy and species delimitation [10,11]. Mechanistically, mitochondrial and nuclear genomes can record different evolutionary histories because they differ in inheritance mode, effective population size, recombination, and exposure to sex-biased or maternally mediated processes [8,12,13]. The mitochondrial genome is maternally inherited and effectively represents a single non-recombining locus, so it can be rapidly displaced by introgression, mitochondrial capture, selective sweeps, or endosymbiont-associated hitchhiking. By contrast, nuclear phylogenies integrate signals across many independently inherited loci and are therefore generally less sensitive to the stochastic or selective history of any single locus [12,13]. In insects, reliance on mitochondrial markers alone may lead to misleading interpretations when introgression or lineage sorting produces topological conflict between mitochondrial and nuclear datasets [14,15,16].

Pygmy grasshoppers of the family Tetrigidae are a diverse group of Orthoptera characterized by small body size, cryptic habits, and remarkable variation in pronotal and body morphology [17,18,19]. Many tetrigid taxa are difficult to identify because diagnostic morphological characters may be subtle, sexually variable, or affected by environmental conditions. Molecular data are therefore expected to contribute substantially to resolving their taxonomy and phylogeny. However, compared with other orthopteran groups such as Acrididae, the mitogenomic resources available for Tetrigidae remain limited and taxonomically uneven [20,21]. Previous studies have generated several tetrigid mitochondrial genomes and used them to infer phylogenetic relationships among selected genera, but many lineages remain unsampled, and the agreement between mitochondrial and nuclear phylogenetic signals has rarely been tested [21,22,23,24].

Recent advances in long-read sequencing provide new opportunities for improving mitogenome assembly and annotation. PacBio HiFi sequencing produces long reads with high per-base accuracy, allowing complete mitochondrial genomes to be recovered from whole-genome sequencing data with reduced ambiguity in repetitive or structurally complex regions [25,26]. This is particularly valuable for detecting lineage-specific insertions, non-coding regions, and potential structural variation that may be missed or misassembled using short-read data. At the same time, whole-genome HiFi datasets also enable the extraction of nuclear single-copy orthologs, providing an opportunity to compare mitochondrial and nuclear phylogenetic histories within the same specimens.

In this study, we assembled and annotated the complete mitochondrial genomes of four tetrigid species, *Zhengitettix transpicula*, *Formosatettix* sp., *Gibbotettix parvipulvillus*, and *Bolivaritettix* sp., using PacBio HiFi sequencing data. These species represent poorly sampled lineages in Tetrigidae, and their mitochondrial genomes provide new resources for comparative mitogenomics and phylogenetic analysis. We first characterized genome size, nucleotide composition, gene arrangement, non-coding regions, and codon usage patterns, and then reconstructed a mitochondrial phylogeny based on 13 protein-coding genes and two rRNA genes. Because the placement of *Z. transpicula* in the mitogenomic tree was inconsistent with expectations based on morphology and previous taxonomy, we used an integrative framework to evaluate this unexpected placement rather than interpreting the mitochondrial topology alone. Specifically, we compared the mitochondrial signal with morphology-based phylogenetic evidence, a published nuclear single-copy ortholog tree, read-based assembly validation, protein-coding gene integrity checks, and NUMT screening against the available nuclear genome. Through this approach, we aimed to determine whether the anomalous mitochondrial placement of *Z. transpicula* was more likely to reflect assembly artefact, NUMT interference, or genuine biological mito–nuclear discordance. By explicitly combining mitochondrial, nuclear, morphological, and assembly-validation evidence, our study expands the mitogenomic dataset of Tetrigidae while also evaluating the power and limitations of mitochondrial genomes in insect taxonomy.

## 2. Materials and Methods

### 2.1. Taxon Sampling and Specimen Preservation

Four pygmy grasshopper samples belonging to Tetrigidae were included in this study: *Zhengitettix transpicula*, *Formosatettix* sp., *Gibbotettix parvipulvillus*, and *Bolivaritettix* sp. The labels *Formosatettix* sp. and *Bolivaritettix* sp. are informal provisional sample labels used only for specimen tracking in the present mitogenomic study. They do not constitute formal species descriptions, establishment of available names, or nomenclatural acts under the International Code of Zoological Nomenclature. Formal taxonomic treatment of these taxa will be provided elsewhere.

Adult specimens were collected from Yunnan and Guangxi, China, between May and August 2024. One female individual of *Z. transpicula* was collected from Dali Bai Autonomous Prefecture, Yunnan Province, China, at approximately 100°13′ E, 25°42′ N and an elevation of approximately 1200 m. The specimen was collected from the edge of shrub vegetation. The other three samples were collected from Yangshuo County, Guilin City, Guangxi Zhuang Autonomous Region, China, at approximately 110°29′ E, 24°47′ N. *Formosatettix* sp. DY2024 was collected from the edge of a forested valley in a hilly area, *G. parvipulvillus* from grassland in a limestone mountain habitat at approximately 300 m elevation, and *Bolivaritettix* sp. from valley grassland near a stream. All specimens were immediately preserved in absolute ethanol in the field and transported to the laboratory. Specimens were stored at −20 °C before DNA extraction. Voucher specimens were deposited in the Insect Collection of Hechi University, Hechi, Guangxi, China.

### 2.2. DNA Extraction and PacBio HiFi Sequencing

Genomic DNA was extracted from thoracic muscle tissue using a modified CTAB protocol. Approximately 50 mg of muscle tissue was dissected from each specimen and ground into powder in liquid nitrogen. The powdered tissue was incubated with 500 μL of preheated CTAB extraction buffer at 65 °C for 1 h. DNA was subsequently purified by sequential extraction with phenol/chloroform/isoamyl alcohol 25:24:1 and chloroform/isoamyl alcohol 24:1. DNA was precipitated using absolute ethanol, washed with 70% ethanol, air-dried, and dissolved in 50 μL of TE buffer.

The concentration and purity of extracted DNA were assessed using a NanoDrop 2000 spectrophotometer Thermo Scientific, Waltham, MA, USA. DNA integrity was evaluated by electrophoresis on a 1% agarose gel. Qualified DNA samples were used for PacBio HiFi sequencing. Sequencing libraries were constructed using the SMRTbell Express Template Prep Kit 2.0 according to the manufacturer’s protocol. Only DNA samples passing both laboratory integrity inspection and the sequencing provider’s library-construction quality control were used for PacBio HiFi sequencing. Sequencing was performed on the PacBio Revio platform by Berry Genomics Co., Ltd. (Nanjing, Jiangsu, China). Approximately 15 Gb of HiFi data were generated for each sample, with an average read length of approximately 15 kb.

### 2.3. Mitochondrial Genome Assembly and Validation

Complete mitochondrial genomes were assembled from PacBio HiFi reads using a reference-guided read extraction followed by de novo assembly strategy. The published mitochondrial genome of *Tetrix japonica* GenBank accession number NC_018543 was used as the initial reference for mitochondrial read extraction. Initially, raw HiFi reads were aligned to the reference mitogenome using minimap2 v2.24 [27] with the map-hifi preset: minimap2-ax map-hifi reference_mt.fa hifi_reads.fq > aln.sam.The resulting SAM files were converted and processed using SAMtools v1.19 and BCFtools v1.19 [28]. Coverage depth was calculated using mosdepth v0.3.3 [29]. For *Formosatettix* sp., *Gibbotettix parvipulvillus*, and *Bolivaritettix* sp., the mitochondrial-read extraction recovered 3806, 4274, and 3894 candidate reads longer than 3000 bp, respectively, corresponding to 69.07 Mb, 67.58 Mb, and 63.67 Mb of candidate mitochondrial read sequence. These values correspond to read-base equivalent mitochondrial coverages of approximately 3842.54×, 4085.68×, and 3769.05× relative to the final mitogenome lengths. For *Zhengitettix transpicula*, the available minimap2 BAM file contained 3988 mapped reads and 64.61 Mb of mapped bases, corresponding to an approximate read-base equivalent coverage of 4264.12× relative to the final mitogenome. Reads mapped to the mitochondrial reference were extracted and converted into FASTA format. The extracted mitochondrial-like reads were then assembled de novo using Flye v2.9.6 [30] with default parameters. Candidate mitochondrial contigs were identified by BLAST v2.16.0 [31] searches against published tetrigid mitochondrial genomes and the NCBI nucleotide database.

To verify the circularity of each mitochondrial genome, candidate contigs were examined for terminal overlap and duplicated mitochondrial genes. Because the draft assemblies contained non-target or graph-expanded sequences substantially longer than a typical insect mitochondrial genome, final mitochondrial sequences were manually curated from the validated mitochondrial contigs. Circular sequences were reoriented to start from the *trnI* gene to facilitate comparison with previously published insect mitogenomes. The four newly assembled mitochondrial genomes were deposited in GenBank under accession numbers PQ869509–PQ869512.

We explicitly screened the final *Z. transpicula* mitogenome for nuclear mitochondrial DNA segments, hereafter NUMT (Nuclear mitochondrial)-like sequences. The final mitochondrial genome sequence was used as a BLASTN query against the *Z. transpicula* nuclear genome assembly also using BLAST v2.16.0 [31]. BLASTN hits were retained using the following criteria: sequence identity ≥ 80%, E-value ≤ 1 × 10^−10^, and alignment length ≥ 100 bp. For each hit, we recorded the nuclear chromosome or scaffold, alignment length, percentage identity, query coordinates, subject coordinates, E-value, bitscore, and percentage of the mitochondrial query covered by the individual hit.

### 2.4. Mitochondrial Genome Annotation and Visualization

The assembled mitochondrial genomes were initially annotated using the MITOS2 (v2.1.10) local server with the RefSeq 89 Metazoa/Arthropoda reference dataset and the invertebrate mitochondrial genetic code. The annotation results were manually inspected and corrected in Geneious Prime 2023.1.2. Protein-coding gene boundaries were checked by comparison with published tetrigid mitogenomes. Start and stop codons were verified according to the invertebrate mitochondrial genetic code.

Transfer RNA genes were identified based on MITOS2 predictions and manually checked according to their secondary structures. Ribosomal RNA gene boundaries were determined by comparison with closely related tetrigid species. The final mitochondrial genome maps were generated using Proksee platform (https://proksee.ca/ (accessed on 5 May 2026)) [32]. Intergenic spacers and overlapping regions were identified based on the final annotation files. Particular attention was given to non-coding regions located between *trnS2* UCN and *nad1*, because this region showed remarkable length variation among the newly assembled mitogenomes. For validation of the mitochondrial assembly of *Z. transpicula*, the independently assembled contig was aligned against the final annotated *Z. transpicula* mitogenome PQ869509 using MAFFT v7.505. Pairwise identity and alignment statistics were calculated in Geneious Prime 2023.1.2.

### 2.5. Taxon Sampling for Mitogenomic Phylogenetic Analysis

The four newly assembled mitogenomes were combined with published tetrigid mitogenomes downloaded from GenBank. Two orthopteran species, *Loxoblemmus doenitzi* and *Gryllus bimaculatus*, were selected as outgroups. Taxon names, GenBank accession numbers, and sequence sources are listed in Supplementary Table S1. Each mitochondrial gene was extracted and organized using PhyloSuite v1.2.2 [33]. The 13 PCGs were aligned individually in codon mode using MAFFT v7.505 [34] implemented in PhyloSuite. The two rRNA genes were aligned separately using MAFFT with the --auto strategy. Poorly aligned regions were inspected manually. Individual gene alignments were concatenated into a single matrix also using PhyloSuite.

Maximum likelihood ML analysis was performed using IQ-TREE v3.1.1 [35]. The best-fitting substitution model was selected by ModelFinder v3.1.1 according to the Bayesian information criterion BIC. Branch support was estimated with 1000 ultrafast bootstrap replicates and 1000 SH-aLRT replicates; Bayesian inference BI analysis was conducted using MrBayes v3.2.7a [36]. Two independent runs with four Markov chains were performed for 10,000,000 generations, with trees sampled every 1000 generations. The first 25% of sampled trees were discarded as burn-in. Convergence was assessed by confirming that the average standard deviation of split frequencies was below 0.01 and that the effective sample size ESS values were greater than 200 in Tracer v1.7.2 [37].

The ML and BI analyses produced identical topologies. Therefore, a single combined mitochondrial tree was presented. Nodal values are shown as ML bootstrap support/BI posterior probability. BI posterior probabilities were converted to percentages for graphical consistency. The final tree was visualized and edited using the R packages ggtree v3.8.2, ggplot2 v3.4.4, and treeio v1.24.3 [38].

### 2.6. Morphological Phylogeny, Nuclear Reference Tree, Topological Comparison, and Assembly Validation

A morphology-based phylogenetic analysis was performed using a matrix of 92 discrete morphological characters. The character list and character-state matrix are provided in Supplementary Table S2. Taxa included in the morphological matrix were selected from available laboratory specimens and published taxonomic materials. Because the morphological sampling was based on specimen availability, it was not designed to correspond exactly to the mitogenomic taxon set.

The morphological matrix was compiled in Mesquite v3.81. Phylogenetic reconstruction was performed in IQ-TREE v3 under the MK + FQ model for discrete morphological characters. All characters were treated as unordered and equally weighted, and missing or inapplicable states were coded as “?”. The best-fitting model parameters were estimated during tree search. Branch support was assessed using 1000 ultrafast bootstrap replicates. The resulting morphology-based tree was visualized using the R packages v4.3.3 ggtree and ggplot2.

The nuclear phylogenomic tree used for comparison was taken directly from our recently published phylotranscriptomic study of tetrigid grasshoppers [17]. This tree was inferred from 1962 genome-wide single-copy orthologous loci and showed 100 bootstrap support at all displayed nodes. The nuclear dataset included representative tetrigid taxa overlapping with the present mitochondrial sampling at the genus or species level, but not all terminals were derived from the same physical individuals sequenced for mitogenomes in the present study. Therefore, the nuclear comparison was used as an independent representative-taxon framework for evaluating broad mito–nuclear conflict rather than as a strict same-individual phylogenomic test. No nuclear phylogenomic reconstruction was repeated in the present study. Topological comparisons among the mitochondrial tree, morphology-based tree, and nuclear single-copy ortholog tree were conducted using tanglegrams. The mitochondrial tree was compared separately with the morphology-based tree and with the published nuclear ortholog tree. Tanglegrams were generated in R using phytools v2.1-1 [39] and further edited with ggplot2. Only taxa shared between each pair of trees, or taxonomically corresponding representative taxa, were connected in the tanglegrams.

## 3. Results

### 3.1. General Features and Structural Variation of the Four Newly Assembled Mitogenomes

**Figure 1:**
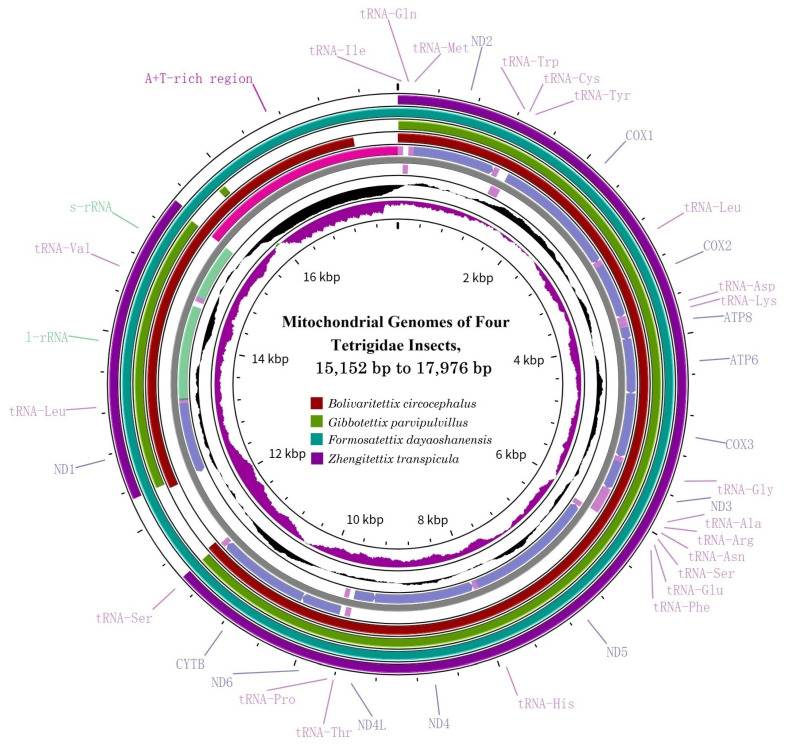
Circular maps of the four newly assembled tetrigid mitochondrial genomes. The four outer colored rings represent the complete mitochondrial genomes of *Bolivaritettix* sp. red, *Gibbotettix parvipulvillus* green, *Formosatettix* sp. cyan, and *Zhengitettix transpicula* purple. The annotated mitochondrial gene map is shown inside the species-specific rings. Protein-coding genes, rRNA genes, tRNA genes, and the A+T-rich region are labeled around the circle. Genes located on different strands are indicated by their orientation on the circular map. The inner plots show GC content and GC skew calculated using a sliding-window approach. The scale inside the circle indicates genome position in kilobases.

Four complete mitochondrial genome sequences were obtained for *Zhengitettix transpicula*, *Formosatettix* sp., *Gibbotettix parvipulvillus*, and *Bolivaritettix* sp. The final curated mitogenomes were each represented by a single sequence. Their lengths were 15,152 bp for *Z. transpicula*, 17,976 bp for *Formosatettix* sp., 16,541 bp for *G. parvipulvillus*, and 16,893 bp for *Bolivaritettix* sp. GC contents were 29.22%, 25.14%, 30.91%, and 25.73%, respectively (Figure 1). All four mitogenomes contained the standard insect mitochondrial gene complement, including 13 protein-coding genes, 22 transfer RNA genes, and two ribosomal RNA genes. The final curated mitogenome lengths differed from the longest intermediate Flye draft contigs. The longest draft contigs ranged from 66.86 kb to 81.02 kb in the intermediate assembly directories, substantially longer than a typical insect mitochondrial genome. These longer draft sequences were therefore not treated as final mitogenomes. Instead, final sequences were curated from mitochondrial contigs based on mitochondrial gene content, expected genome size, sequence composition, and downstream validation.

Candidate mitochondrial read extraction recovered substantial read support for all four mitogenomes. For *Formosatettix* sp., 3806 reads longer than 3000 bp were retained, with a total candidate mitochondrial read length of 69.07 Mb. Relative to the final 17,976 bp mitogenome, this corresponds to an approximate read-base equivalent coverage of 3842.54×. For *G. parvipulvillus*, 4274 reads longer than 3000 bp were retained, totaling 67.58 Mb and corresponding to approximately 4085.68× coverage relative to the final 16,541 bp mitogenome. For *Bolivaritettix* sp., 3894 reads longer than 3000 bp were retained, totaling 63.67 Mb and corresponding to approximately 3769.05× coverage relative to the final 16,893 bp mitogenome.

The gene arrangement of the four newly assembled mitogenomes was identical to that of previously reported tetrigid mitogenomes, indicating a conserved mitochondrial gene order within Tetrigidae. Most genes were encoded on the majority strand, whereas four PCGs, namely *nad1*, *nad4*, *nad4L*, and *nad5*, were located on the minority strand. This strand distribution is consistent with the typical mitochondrial organization reported in orthopteran insects. No gene rearrangement, gene loss, or duplicated mitochondrial gene was detected in the four newly assembled genomes.

The nucleotide composition of the four mitogenomes showed a strong A+T bias, with A+T contents ranging from 69.1% in *G. parvipulvillus* to 74.9% in *Formosatettix* sp. The A+T content was 70.0% in *Z. transpicula* and 74.3% in *Bolivaritettix* sp. All four mitogenomes exhibited positive AT skew values 0.101–0.212 and negative GC skew values −0.311 to −0.231, suggesting an asymmetric nucleotide composition between the two mitochondrial strands. Among the four species, *Z. transpicula* showed the highest AT skew 0.212, whereas *Formosatettix* sp. showed the lowest AT skew 0.101. The GC skew was most negative in *G. parvipulvillus* −0.311 and least negative in *Formosatettix* sp. −0.231. These patterns are generally consistent with the nucleotide compositional bias observed in other tetrigid mitogenomes.

**Figure 2:**
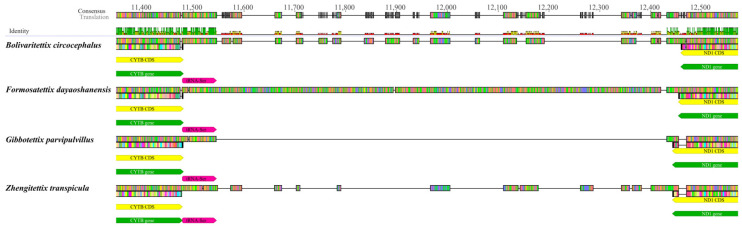
Alignment of the *cytb*–*trnS2* UCN–*nad1* region among the four newly assembled tetrigid mitochondrial genomes. The alignment shows the local mitochondrial region spanning the end of *cytb*, *trnS2* UCN, and the beginning of *nad1*. Gene annotations are displayed below each sequence: yellow arrows indicate coding sequences CDSs, green arrows indicate annotated genes, and pink arrows indicate *trnS2*.

Although the overall gene order was conserved, conspicuous length variation was detected in the region between *trnS2* UCN and *nad1*. Three of the four newly assembled mitogenomes contained relatively long non-coding intergenic regions in this position Figure 2. The lengths of these regions were 270 bp in *Z. transpicula*, 895 bp in *Formosatettix* sp., and 353 bp in *Bolivaritettix* sp. By contrast, no comparable long insertion was detected in *G. parvipulvillus*, in which *trnS2* and *nad1* were separated by only a short intergenic interval. The 895 bp region in *Formosatettix* sp. represents the longest non-coding insertion identified among the four newly sequenced mitogenomes and accounts for its larger genome size relative to the other three species.

The non-coding regions between *trnS2* and *nad1* were characterized by high A+T content and showed no obvious conserved sequence block shared among the three species. BLAST searches did not identify significant similarity to known mitochondrial control regions, annotated mitochondrial genes, or other orthopteran mitochondrial sequences. We also inspected these regions for obvious internal similarity, repeated motifs, and duplicated gene-like fragments, but no clear tandem-repeat structure or conserved repeat unit explaining the length expansion was detected. No additional tRNA gene was predicted within these regions by MITOS2, and manual inspection did not reveal clear evidence of functional gene duplication. Therefore, based on the present sequence-similarity and annotation evidence, these regions are more likely to represent lineage-specific non-coding expansions than secondary control regions or duplicated mitochondrial genes.

To further evaluate whether these regions represented authentic mitochondrial sequences rather than assembly artefacts, the corresponding sequences were checked against the original HiFi reads. Reads spanning the flanking *trnS2*–*nad1* region supported the continuity of the assembled mitochondrial contigs, indicating that these insertions are not likely to be caused by simple misassembly. Therefore, the observed length variation probably reflects lineage-specific structural plasticity in tetrigid mitochondrial genomes. This structurally variable region may represent a useful marker for future comparative mitogenomic and population-level studies in Tetrigidae, although its evolutionary origin and potential functional significance require further investigation.

### 3.2. Mitochondrial and Morphological Phylogenies Reveal Strong Discordance in the Placement of Zhengitettix transpicula

**Figure 3:**
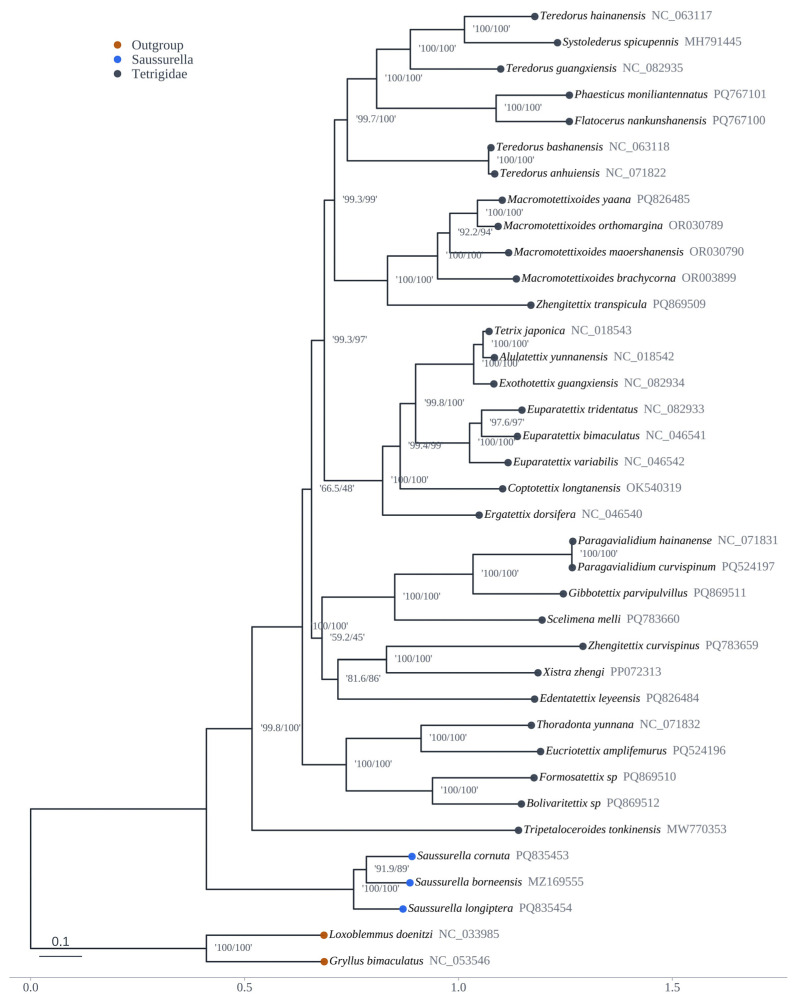
Mitogenomic phylogeny of Tetrigidae inferred from concatenated mitochondrial genes. The phylogeny was reconstructed using the concatenated mitochondrial dataset including 13 protein-coding genes and two rRNA genes. Maximum likelihood ML and Bayesian inference BI analyses produced identical topologies; therefore, only one combined tree is shown. Numbers at nodes indicate ML bootstrap support and BI posterior probability, respectively.

Phylogenetic analyses based on the mitochondrial dataset produced identical topologies under maximum likelihood and Bayesian inference (Figure 3). The two outgroup species, *Loxoblemmus doenitzi* and *Gryllus bimaculatus*, were recovered outside Tetrigoidea with maximal support. Several tetrigid terminal pairs or small clades were strongly supported, including *Teredorus hainanensis* + *Systolederus spicupennis*, *Teredorus bashanensis* + *T. anhuiensis*, *Phaesticus moniliantennatus* + *Flatocerus nankunshanensis*, *Tetrix japonica* + *Alulatettix yunnanensis*, *Euparatettix tridentatus* + *E. bimaculatus*, and *Paragavialidium hainanense* + *P. curvispinum*. Among the newly sequenced taxa, *G. parvipulvillus* was recovered close to *Paragavialidium* and *Scelimena*, whereas *Formosatettix* sp. and *Bolivaritettix* sp. formed a strongly supported sister pair. *Zhengitettix transpicula* was not recovered close to *Zhengitettix curvispinus*. Instead, it was placed near the *Macromotettixoides* lineage in the mitochondrial topology.

**Figure 4:**
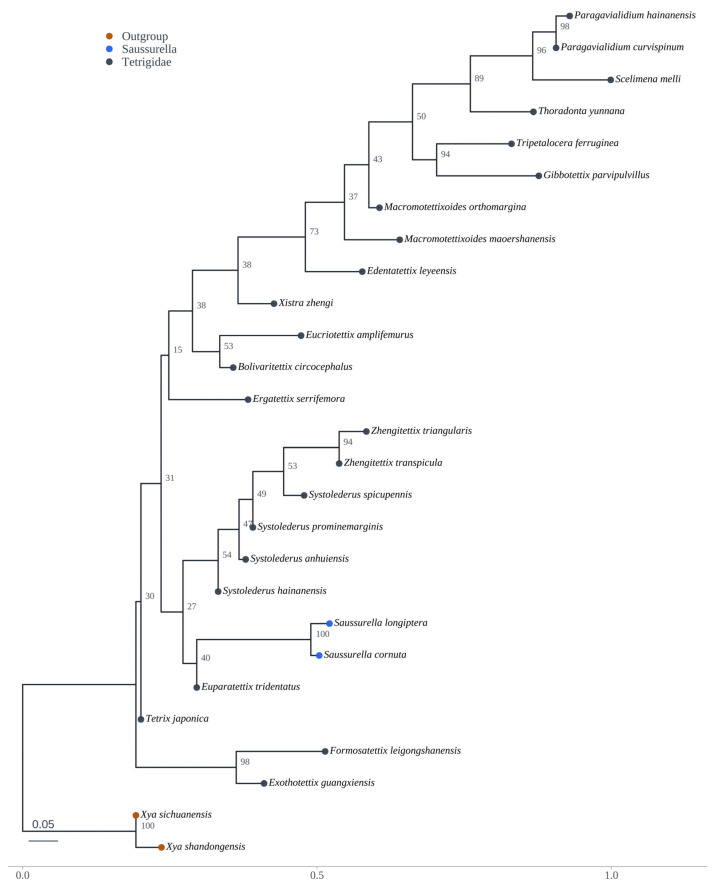
Morphology-based phylogenetic tree inferred from 92 morphological characters. Numbers at nodes indicate bootstrap support values.

The morphology-based analysis of 92 discrete characters yielded a different placement for *Z. transpicula* (Figure 4). In the morphological tree, *Z. transpicula* was recovered as sister to *Z. triangularis* with bootstrap support of 94. Thus, the morphological topology supported a *Zhengitettix*-like placement of *Z. transpicula*, whereas the mitochondrial topology placed it near *Macromotettixoides*.

### 3.3. Cross-Dataset Comparison and Validation of the Mitochondrial Placement of Zhengitettix transpicula

The mitochondrial topology was compared with the morphology-based tree and with the published nuclear single-copy ortholog tree using predefined terminal correspondences. The nuclear tree was based on 1962 single-copy orthologous genes from Ref. [15] and all displayed nuclear nodes received 100% bootstrap support. The comparison was therefore used as an independent framework for evaluating whether the mitochondrial placement of selected taxa agreed with non-mitochondrial evidence.

**Figure 5:**
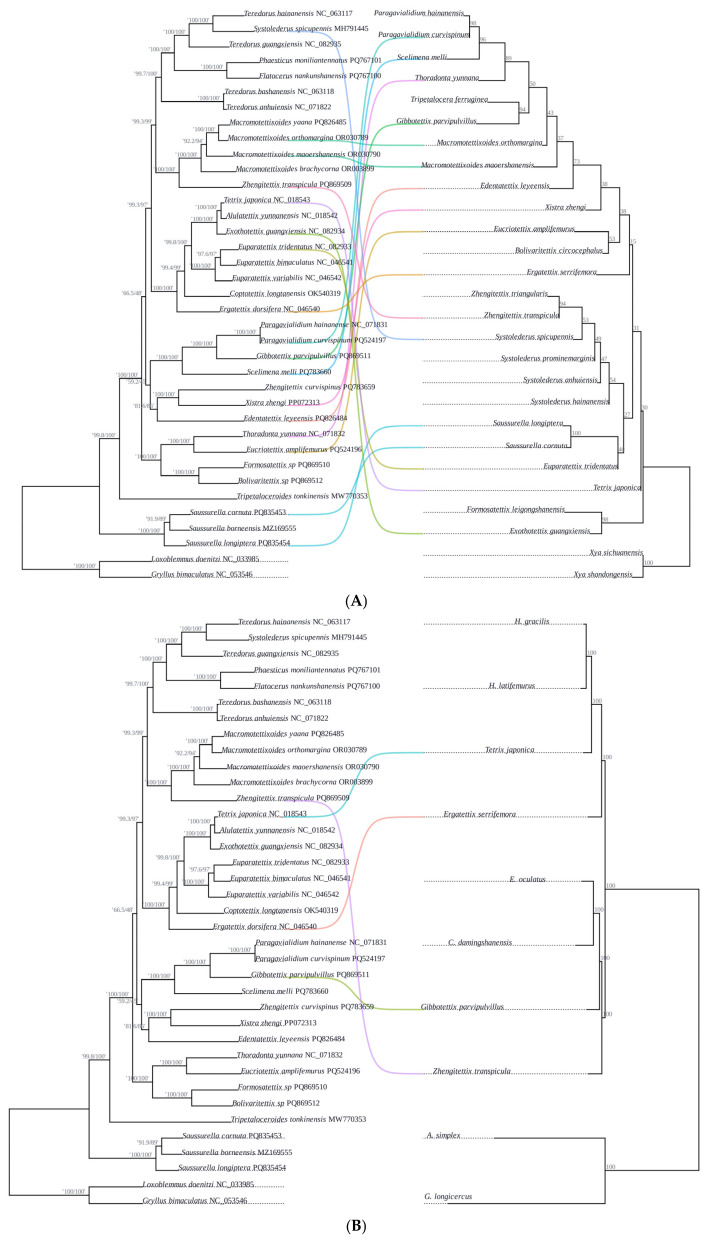
Comparisons between the mitochondrial topology and independent morphological and nuclear genomic phylogenies. (**A**), Tanglegram comparison between the mitogenomic tree and the morphology-based tree inferred from 92 morphological characters. The left tree represents the mitochondrial topology, and the right tree represents the morphological topology. Colored lines indicate predefined taxon-correspondence groups used for visual comparison, each genus was assigned a dependent color. (**B**), Tanglegram comparison between the mitogenomic tree and the nuclear phylogeny inferred from genome-wide single-copy orthologs. The left tree represents the mitochondrial topology, and the right tree represents the nuclear ortholog topology. All displayed nodes in the nuclear ortholog tree received 100 bootstrap support, indicating a highly stable nuclear genomic signal.

The tanglegram comparing mitochondrial and morphological trees showed several topological differences across Tetrigidae (Figure 5A). Among the focal taxa, the most prominent difference involved *Z. transpicula*. This taxon was placed near *Macromotettixoides* in the mitochondrial tree but was linked with *Z. triangularis* in the morphology-based tree.

The comparison between the mitochondrial tree and the nuclear single-copy ortholog tree also showed disagreement in the placement of the *Zhengitettix* lineage (Figure 5B). The nuclear topology did not support association of *Zhengitettix* with *Macromotettixoides*. Instead, the nuclear placement of *Zhengitettix* was more consistent with the region represented by *Z. curvispinus* in the mitochondrial topology.

Compared with *Z. transpicula*, the other newly sequenced taxa showed less direct conflict with expected generic placement. *Formosatettix* sp. and *Bolivaritettix* sp. formed a strongly supported pair in the mitogenomic tree. *G. parvipulvillus* showed some positional differences among comparative frameworks, but these did not involve the same direct contradiction between mitochondrial, morphological, and nuclear evidence observed for *Z. transpicula*.

**Figure 6:**
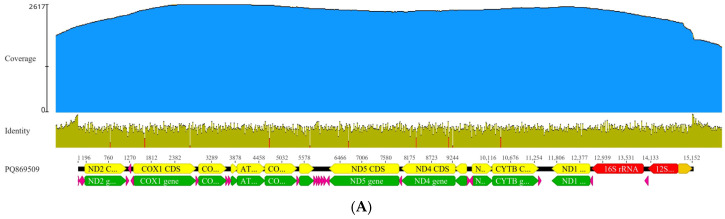

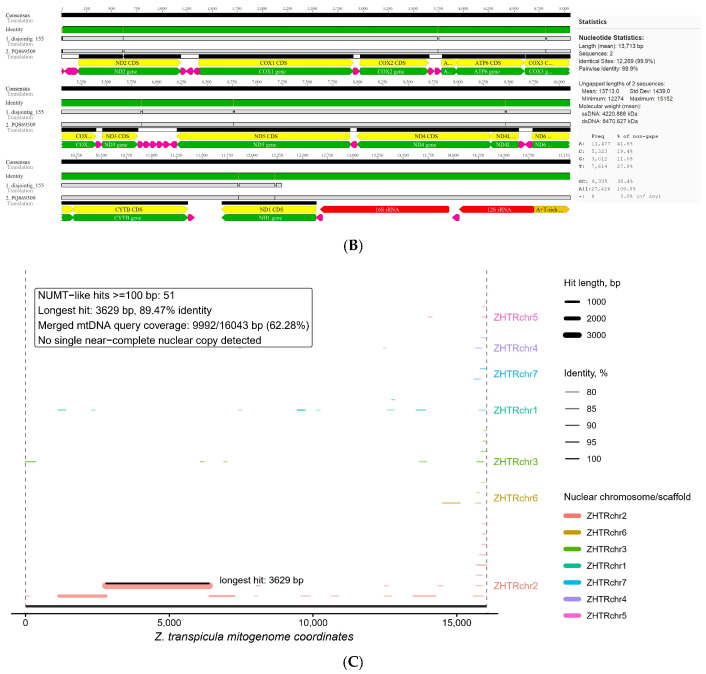
Validation of the *Zhengitettix transpicula* mitochondrial genome assembly. (**A**) HiFi read-depth profile across the final *Z. transpicula* mitogenome. The blue track shows site-wise coverage along the mitochondrial coordinates, and the lower tracks show local sequence identity and annotated mitochondrial genes. (**B**) Alignment between an independently assembled mitochondrial contig from the original HiFi data and the final annotated mitogenome PQ869509. (**C**) BLASTN-based screening of the final *Z. transpicula* mitogenome against the chromosome-level nuclear genome assembly. NUMT-like hits with identity ≥ 80%, E-value ≤ 1 ×10^−10^, and alignment length ≥ 100 bp are projected onto mitochondrial coordinates.

The final *Z. transpicula* mitochondrial genome was validated using HiFi read mapping, independent contig comparison, and NUMT screening. The curated mitogenome was 15,152 bp long with a GC content of 29.22%. The minimap2 BAM file contained 3988 mapped reads and 64.61 Mb of mapped bases, corresponding to an approximate read-base equivalent coverage of 4264.12× when total mapped bases were divided by the mitogenome length. This value should be interpreted as a theoretical read-base estimate rather than uniform per-site depth, because many mapped HiFi reads covered only part of the mitochondrial genome. Site-wise coverage along the mitochondrial coordinates was therefore lower and variable, with a mean depth of 1111.2×, a standard deviation of 1070.5 and a maximum depth of 2617× (Figure 6A). No abrupt coverage break, extended zero-coverage region, or sharply isolated low-coverage interval was observed, indicating that the assembly was consistently supported by the original long-read data. An independently assembled mitochondrial contig from the original HiFi data was also aligned to the final annotated mitogenome PQ869509. The two sequences showed 99.9% pairwise identity, with 12,269 identical sites and eight gap positions in the aligned region (Figure 6B). This high similarity provides independent support for the final mitochondrial assembly.

BLASTN screening of the final *Z. transpicula* mitogenome against the chromosome-level nuclear genome assembly identified 51 NUMT-like hits longer than 100 bp under the criteria of identity ≥ 80% and E-value ≤ 1 ×10^−10^. These hits were distributed across seven nuclear chromosomes or scaffolds and ranged from 102 bp to 3629 bp in length, with identities of 80.00–98.12%. The longest hit, located on ZHTRchr2, covered 3629 bp of the mitochondrial query with 89.47% identity, corresponding to 22.62% of the mitogenome. Merged mitochondrial query intervals covered 9992/15152 bp, or 65.94%, of the mitogenome, but this coverage resulted from multiple fragmented nuclear matches rather than a single near-complete nuclear mitochondrial copy (Figure 6C). No individual nuclear hit approached the full mitogenome length, and no complete mitogenome-like nuclear locus was detected. These results indicate that although fragmented NUMT-like sequences are present in the *Z. transpicula* nuclear genome, the final mitochondrial assembly is unlikely to represent a single nuclear mitochondrial pseudogene.

## 4. Discussion

Mitochondrial genomes have become indispensable in insect systematics because they are compact, abundant in sequencing libraries, and relatively straightforward to assemble and annotate compared with nuclear genomes [2,4,5]. The four complete mitogenomes generated here add new genomic resources for Tetrigidae, a family in which molecular sampling remains uneven despite its considerable taxonomic diversity and morphological complexity [20,21,23,24,40,41]. All four mitogenomes retained the typical insect mitochondrial gene complement of 13 protein-coding genes, 22 tRNAs, and two rRNAs, and no gene rearrangement was detected. This conserved organization agrees with the general pattern reported for Orthoptera and previously sequenced tetrigid mitogenomes [3,4,23]. The overall structural conservatism of these genomes reinforces the value of mitogenomes as comparative markers in tetrigid systematics, particularly for poorly sampled genera.

Although the gene order was conserved, the four genomes differed substantially in length, ranging from 15,152 bp in *Z. transpicula* to 17,976 bp in *Formosatettix* sp. This difference was mainly associated with lineage-specific non-coding regions between *trnS2* and *nad1*. Similar length variation in insect mitogenomes is often concentrated in non-coding regions, including the control region and lineage-specific intergenic spacers [21,23,24]. In the present data, the *trnS2*–*nad1* region varied markedly among the four species, with conspicuous insertions in *Formosatettix* sp., *Bolivaritettix* sp., and *Z. transpicula*, but not in *G. parvipulvillus*. The lack of clear sequence conservation among these regions suggests rapid evolution rather than retention of an ancient conserved element. Because no additional tRNA or duplicated protein-coding gene was detected, these sequences are unlikely to represent functional gene duplications. They may instead correspond to lineage-specific intergenic expansions, degraded duplicated fragments, or neutrally evolving non-coding insertions. Comparable non-coding expansions have been reported in other insect mitochondrial genomes and may contribute useful markers for closely related taxa when coding genes are too conserved or phylogenetically saturated.

The use of PacBio HiFi data was important for interpreting these structural variants. Long and highly accurate reads reduce ambiguity in regions that are difficult to resolve with short-read sequencing, especially repetitive or AT-rich non-coding segments [25,26,42]. The HiFi read support across the variable *trnS2*–*nad1* regions argues against simple assembly artifacts and suggests that these insertions represent genuine mitochondrial features. This is relevant for comparative mitogenomics because unusual non-coding regions are sometimes dismissed as assembly errors when they are identified from short-read assemblies alone. Long-read data therefore not only improve genome completeness but also make it possible to evaluate whether unexpected mitochondrial structures are authentic. In this sense, the present study illustrates how newer sequencing technologies can strengthen classical mitogenomic work by increasing confidence in both gene annotation and structural interpretation.

The mitochondrial phylogeny inferred from concatenated protein-coding and rRNA genes was well resolved across many terminal relationships, and ML and BI analyses produced identical topologies. Several relationships were strongly supported, including species pairs or small clades such as *Paragavialidium hainanense* + *P. curvispinum*, *Formosatettix* sp. + *Bolivaritettix* sp., and multiple *Teredorus*, *Euparatettix*, and *Macromotettixoides* lineages. These results confirm that mitochondrial genomes provide substantial phylogenetic signal for many tetrigid relationships. Similar conclusions have been reached in broader tetrigidae studies, where complete mitochondrial datasets often improve resolution relative to single-gene markers [21,43]. Nevertheless, strong support in a mitochondrial tree does not automatically imply agreement with the species tree. Because the mitochondrial genome is inherited as a single non-recombining locus, it reflects one genealogical history and may be strongly affected by stochastic lineage sorting, introgression, selective sweeps, demographic asymmetry, or sex-biased dispersal [7,8,9,44].

The mitochondrial placement of *Z. transpicula* conflicts with both morphology and the published nuclear phylogenomic framework. In the mitochondrial tree, *Z. transpicula* was not recovered close to other *Zhengitettix* representatives, whereas the morphology-based analysis placed it with *Zhengitettix*-like taxa and the nuclear single-copy ortholog tree did not support a close relationship between *Zhengitettix* and *Macromotettixoides*. Because the *Z. transpicula* mitogenome is supported by independent assembly evidence and because the nuclear genome lacks a near-complete mitochondrial copy that would explain the assembly as a single NUMT, the discordance is unlikely to be caused solely by a simple assembly artifact [45].

This pattern is best interpreted as a case of cyto-nuclear and cyto-morphological discordance. Such discordance is not exceptional in animal systematics and has been documented across many groups [7,8,9,44]. Although several mechanisms can generate mito–nuclear discordance, the present pattern is more consistent with mitochondrial capture than with a purely stochastic explanation. Both the mitochondrial and non-mitochondrial signals are strongly supported, but they support different placements of *Z. transpicula*. If incomplete lineage sorting were the primary cause, one might expect conflict to be more diffuse across closely related lineages or to involve uncertainty in both mitochondrial and nuclear relationships. Instead, the nuclear and morphological evidence consistently support a *Zhengitettix*-like affinity, whereas the mitochondrial genome alone is displaced toward the *Macromotettixoides* region. This pattern is compatible with an ancient introgression event followed by backcrossing, in which the nuclear genome and morphology retained the recipient-lineage identity while the mitochondrial haplotype was replaced [10,11,16]. Incomplete lineage sorting is another possible mechanism, especially if relevant lineages diverged rapidly or retained ancestral mitochondrial polymorphism [46,47,48]. Selective processes may also contribute, because mitochondrial haplotypes can spread through populations under selection, sometimes independently of nuclear genomic background [49,50]. In insects, additional processes such as endosymbiont-associated selective sweeps may affect mitochondrial genealogies [49,50,51], although this possibility was not tested here. Distinguishing among these alternatives will require population-level sampling, broader nuclear genomic comparisons, and possibly screening for maternally inherited symbionts.

Among the possible explanations, mitochondrial capture following historical introgression appears most consistent with the present evidence. The mitochondrial topology places *Z. transpicula* near *Macromotettixoides*, whereas both morphology and the nuclear single-copy ortholog framework support a non-*Macromotettixoides* affinity for *Zhengitettix*. This asymmetry, in which the mitochondrial genome alone shows the anomalous placement while non-mitochondrial evidence remains consistent with the traditional generic affinity, is a typical pattern expected after mitochondrial introgression and subsequent backcrossing. Incomplete lineage sorting cannot be excluded, but it is less directly supported here because the conflicting mitochondrial placement is strongly resolved and is not mirrored by the broader nuclear signal. From a taxonomic perspective, these results do not support transferring *Z. transpicula* toward *Macromotettixoides* based on mitochondrial data alone. The morphology-based placement of *Z. transpicula* with *Zhengitettix*-like taxa, together with the published nuclear framework, suggests that the mitochondrial signal is likely misleading with respect to generic affinity. We therefore retain the current taxonomic interpretation of *Z. transpicula* and regard its mitochondrial position as evidence of cyto-nuclear discordance rather than as a basis for taxonomic rearrangement. This case illustrates that mitochondrial trees in Tetrigidae should be used cautiously for genus-level classification unless supported by nuclear and morphological evidence.

Technical error also had to be considered because unexpected mitochondrial placements can result from contamination, chimeric assembly, nuclear mitochondrial insertions, or incorrect sequence reconstruction. Several lines of evidence argue against this explanation for *Z. transpicula*. First, the mitochondrial genome was recovered from high-accuracy HiFi reads rather than from short-read data alone. Second, the independently extracted mitochondrial contig from the original HiFi reads aligned almost perfectly with the final annotated mitogenome, with 99.9% pairwise identity across the compared region. Third, no large-scale structural discrepancy or gene-order conflict was observed in the validation alignment. These results indicate that the mitogenome sequence used for phylogenetic reconstruction is well supported by the original sequencing data. The conflict therefore cannot be reduced to a simple assembly artifact. Instead, the discordance appears to be biological or taxonomic in nature.

The three other newly sequenced species provide useful context. *Formosatettix* sp. and *Bolivaritettix* sp. formed a strongly supported pair in the mitochondrial tree, and their placement did not generate the same level of contradiction among datasets. *G. parvipulvillus* showed some positional differences among comparative trees, but these did not directly challenge its generic assignment in the way observed for *Z. transpicula*. Thus, the discordance is not a general consequence of poor phylogenetic signal in the dataset. Rather, *Z. transpicula* represents a focal case in which mitochondrial signal is strong but inconsistent with independent morphology and nuclear evidence. This distinction is important because weakly supported conflicts are common in phylogenetics, whereas strongly supported conflict among genomes is more informative and often points to complex evolutionary history [1,4,5].

These findings have direct implications for tetrigid taxonomy. Tetrigidae contains many small-bodied, morphologically variable taxa, and diagnostic characters can be subtle or affected by convergence and ecological adaptation [20,24,43]. Mitochondrial genomes are therefore attractive for supplementing morphology, especially in groups with limited molecular data. However, the present study shows that mitogenomic evidence should not be treated as taxonomically decisive when it conflicts with morphology and nuclear genes. For *Zhengitettix*, the nuclear ortholog tree is currently the most reliable independent phylogenetic framework because of its genome-wide scale and uniformly high support. The position of *Zhengitettix* inferred from nuclear data is compatible with the mitochondrial position of *Z. curvispinus* [43], but not with the mitochondrial position of *Z. transpicula*. This suggests that *Z. transpicula* should not be transferred or reinterpreted solely on the basis of its mitochondrial placement. A conservative taxonomic treatment is therefore warranted until broader sampling of *Zhengitettix*, *Macromotettixoides*, and related genera is available.

However, several limitations should be considered when interpreting the present results. First, each newly sequenced taxon was represented by a single individual, preventing assessment of intraspecific mitochondrial variation, heteroplasmy, and population-level robustness. Second, although we optimized the assembly workflow and applied multiple validation steps, mitochondrial genome recovery from whole-genome HiFi reads can be complicated by non-target contigs, repetitive regions, high-copy nuclear fragments, and NUMT-like sequences. Indeed, assembly screening recovered numerous long non-mitochondrial contigs in addition to the target mitochondrial contigs, indicating that off-target sequences may interfere with mitochondrial contig identification. Finally, the present dataset cannot determine whether the discordant mitochondrial placement of *Z. transpicula* reflects incomplete lineage sorting, introgression, mitochondrial capture, selection, endosymbiont-associated replacement, or population-level effects. These alternatives require broader population sampling, same-individual mito-nuclear genomic comparisons, and explicit tests for introgression, selection, and NUMT contamination.

In conclusion, the four newly assembled mitogenomes expand the genomic resources available for Tetrigidae and reveal previously underappreciated structural variation in the *trnS2*–*nad1* region. The mitochondrial phylogeny provides strong resolution for many relationships but places *Zhengitettix transpicula* in a position that conflicts with both morphology and nuclear single-copy orthologs. Independent validation using original HiFi reads confirms that this conflict is unlikely to result from mitochondrial assembly error. The most plausible interpretation is genuine mitochondrial–nuclear discordance, potentially caused by introgression, mitochondrial capture, incomplete lineage sorting, or unresolved taxonomic complexity. Future work should increase taxon sampling within *Zhengitettix* and related genera, incorporate multiple individuals per species, and use genome-wide nuclear data to test the origin of the discordant mitochondrial signal. Such integrative approaches will be essential for converting mitogenomic resources into reliable taxonomic and evolutionary conclusions in Tetrigidae and other insect groups.

## Data Availability

The four newly assembled mitochondrial genomes generated in this study have been deposited in GenBank under accession numbers PQ869509–PQ869512. The raw sequencing data used for mitochondrial genome assembly have been deposited in Figshare and are available at https://doi.org/10.6084/m9.figshare.32204700.

## Supplementary Materials

The following supporting information can be downloaded at: https://www.mdpi.com/article/10.3390/life16061015/s1, Table S1. Taxon sampling and sequence information used in the mitogenomic phylogenetic analysis. Table S2. Morphological character list and character-state matrix used for the morphology-based phylogenetic analysis.
