# Supplementary material for: HiFi-Assembled Mitogenomes of Four Pygmy Grasshoppers Reveal Mito–Nuclear Discordance in Zhengitettix transpicula and Lineage-Specific Mitochondrial Intergenic Length Variation
Source: Life (Basel). 2026 Jun 17;16(6):1015. doi: 10.3390/life16061015 (PMC13301886; doi:10.3390/life16061015)
Supplement: Supplementary file 1 [file life-16-01015-s001.zip › Table S2.pdf]

Morphological Code Matrix for Tetrigidae

92 adult external morphological characteristics were screened, including 2 body characteristics, 3 antennal characteristics, 17 head characteristics, 40 pronotal characteristics, 4 wing characteristics, 20 leg characteristics, 4 genital characteristics, and 2 body color characteristics. Different states of the characteristics are sequentially coded as "0, 1, 2,......". All character states were treated as equally weighted and unordered; "?" represents the character state was not observed, and "-" represents the character is not applicable. Character descriptions and state codings are as follows:

0. Body size: Small (pronotum length < 10.0 mm) (0); Medium (10.0 mm ≤ pronotum length ≤ 20.0 mm) (1); Large (pronotum length > 20.0 mm) (2).
1. Body shape: Slender and long (0); Slender and short (1); Robust (2); Broad and long (3).
2. Antenna shape: Filiform (0); Ovate (1); Triangular in cross-section (2); Moniliform (3).
3. Number of antennal segments: 7 segments (0); 12 segments (1); 14-16 segments (2); 20-22 segments (3); 10 segments (4).
4. Antennal insertion position: Located at the lower third of the anterior margin of the compound eye (0); Located between the lower margins of the compound eyes (1); Located slightly below the lower margin of the compound eye (2); Located far below the lower margin of the compound eye (3).
5. Face in lateral view: Very sloping and protruding forward (0); Not sloping (1).
6. Vertex in dorsal view: Acutely angular, distinctly protruding in front of the compound eyes (0); Rectangular, distinctly protruding in front of the compound eyes (1); Length of the projecting part of the vertex in front equals the dorsal length of the compound eye (2); Length of the projecting part of the vertex in front exceeds half the dorsal length of the compound eye (3); Not or slightly projecting (4).
7. Anterolateral parts of vertex: Sharply toothed, projecting forward (0); Normal (1).
8. Shape of anterior margin of vertex: Straight (0); Arcuate (1); Angular (2); Centrally arcuately projecting and laterally slightly concave (3).
9. Width of vertex compared to width of compound eye: Equal to the width of one eye

(0); Narrower than the width of one eye (1); Wider than the width of one eye (2); Anterior part of vertex extremely narrow (3).

10. Head height: Not exserted above pronotal surface (0); Distinctly exserted above pronotal surface (1).
11. Compound eye height: Not projecting above the level of the pronotum (0); Projecting above the level of the pronotum (1).
12. Distance between compound eyes: Not close together (0); Extremely close together (1).

13. Lateral margins of vertex in frontal view: Not higher than compound eyes (0); Higher than compound eyes (1); Lateral margins absent (2);

14. Vertex foveola in frontal view: Shallow (0); Distinctly U-shaped (1); Distinctly V-shaped (2); Vertex foveola absent (3).

15. Facial carina between antennae: Not broadly scutellate (0); Greatly expanded forming a relatively broad scutellum (1); Facial carina absent (2)

16. Width of longitudinal furrow of frontal costa compared to width of antennal socket: Equal to the width of the antennal socket (0); Narrower than the antennal socket (1); Wider than the antennal socket (2).

17. Vertex in lateral view: Not stepped (0), Two -step-form (1).

18. Frontal costa before the eyes in lateral view: Visible (0); Not visible (1); Extremely prominent (2).

19. Frontal ridge and vertex forming: Obtuse-angled or obtusely rounded (0); Rounded (1); Acute-angled (2);

20. Frontal costa between the eyes in lateral view: Not concave (0); Concave (1).

21. Position of lateral ocelli: Located at the middle of the anterior margin of the compound eye (0); Located at the lower margin of the compound eye (1), Located at the upper 1/3 of the anterior margin of the compound eye (2).

22. Pronotum length: Not reaching abdomen (0); Covering abdomen (1).

23. Pronotal surface: With coarse wrinkles and tubercles (0); Smooth or with small granules (1); With fine wrinkles (2); With numerous irregular concavities (3).

24. Pronotum tectiform: No (0); Slightly (1); Strongly roof-like (2).

25. Pronotum compression: Extremally laterally compressed, sides steeply sloping downwards, dorsum arched upwards (0); Not laterally compressed (1).
26. Pronotum in anterior half: Normal or low (0); Distinctly raised and humpbacked (1).
27. Upper margin of pronotum between humeral angles in lateral view: Low (0); Slightly raised (1); Strongly raised, hump-like (2); Undulating (3).
28. Shape of hump between humeral angles of pronotum: Triangular (0); Arcuate (1); Undulate (2); Hump absent (3).
29. Median carina of pronotum before humeral angles: Not elevated (0); Slightly lamellate (1); Distinctly lamellately elevated (2).
30. Dorsal margin of pronotum, lateral view: Straight (0); Wavy (1); Slightly arcuate (2); Extremely highly arched (3); Slightly elevated anterior to humeral angles (4).
31. Dorsal margin of pronotum anterior to humeral angles, lateral view: Without tooth or notch (0); With a tooth notch on the elevation (1).
32. Pronotum dorsum near the middle: With a strong triangular spine (0); With a distinct concavity/depression (1).
33. Humeral angles: Absent (0); Arched (1); Angular (2).
34. Apex of hind pronotal process: With a central notch (0); Pointed (1); Broadly rounded (2).
35. Anterior part of pronotum, lateral view: Not projecting beyond head (0); Angularly projecting above head (1); Arcuately projecting above head (2).
36. Pronotal cornu: extended forwards (0); Obliquely upwards (1).
37. Anterior projection of pronotum, lateral view: Straight (0); Apex curved downward (1).
38. Pronotum between posterior sulcus and humeral angles: Forming a four-nodule-like elevation (0); Not forming a four-nodule-like elevation (1).
39. Median carina of pronotum posterior to humeral angles: Undulating, a series of connected small tubercles (0); Not a series of connected small tubercles (1).
40. Head: Short and not retracted into pronotum (0); Retracted into pronotum (1).
41. Anterior part of pronotum expanded: No (0); Yes (1).
42. Apex of posterior angle of pronotal lateral lobe: Rounded (0); Truncate (1); Spinose

(2); Angular (3).

43. Pronotal lateral lobes: Produced outwards (0); Directed downward (1).

44. Spine of pronotal lateral lobe posterior angle: Directed laterally (0); Directed backward (1); Curved forward (2).

45. Anterior margin of pronotum: Normal (0); With a row of tooth-like projections (1).

46. Apex of pronotal lateral lobe: With 5 sharp teeth (0); Without 5 sharp teeth (1).

47. Junction of humeral apex ridge and lower margin of pronotum: Located anterior to the middle of the lower margin of pronotum (0); Located at or posterior to the middle of the lower margin of pronotum (1).

48. Center of pronotum anterior margin: Normal (0); With a finger-like process (1).

49. Margins of pronotum at humeral angles: Smooth (0); With coarse tubercles (1).

50. Lateral carinae of pronotum posterior to humeral angles: Undulating (0); Straight (1).

51. Median carina of pronotum in prozona: Undulating and lamellately elevated (0); Straight and low (1).

52. Between humeral angles of pronotum: With swollen tubercle 0; Without tubercle (1).

53. Pronotum posterior to humeral angles: Flat (0); Concave/Depressed (1).

54. Shape of pronotum anterior margin: Straight (0); Arcuate (1); Obtusely angled (2); Acutely angled (3); Slightly concave in the middle (4).

55. Pronotum anterior margin beind the eyes with frontolateral projections: No (0); Yes (1).

56. Median carina of pronotum: Not lamellate or slightly lamellate (0); Lamellate anteriorly and flat posteriorly (1); Fully lamellately elevated for entire length (2).

57. Median carina of pronotum complete for entire length: Yes (0); No (1).

58. Lateral carinae of pronotum in prozona: Parallel (0); constricted backwards (1); Absent (2)

59. Interhumeral carina of pronotum: Absent (0); 1 pair present (1); 2 pairs present (2).

60. Posterior margins of pronotal lateral lobes: With ventral sinus and tegminal (upper) sinus (0); Only with ventral sinus (1).

61. Ventral margin of posterior process of pronotum: Straight (0); Curved downward (1).

62. Position of tegmina: On dorsal side of body (0); On lateral sides of body (1).

63. Position of hind wings or hind wing buds: Beneath forewings (0); Beneath pronotum (1)

64. Tegmina: Normal (0); Small, reduced (1); Absent or not visible (2).

65. Hind wings: Normal (0); Small, reduced (1); Absent or not visible (2).

66. Foreleg: Fossorial (digging) (0); Cursorial (walking) (1).

67. Upper side of fore femur: With shallow longitudinal groove (0); Without groove (1).

68. Lower margin of fore femur: Straight (0); Wavy (1); With tooth-like projections (2).

69. Lower margin of mid femur: Straight (0); Wavy (1); With 1 tooth-like projection (2); With 2 tooth-like projections (3).

70. Fore and mid femora: Not covered with long hairs (0); Densely covered with long hairs (1).

71. Apex of mid femur: Without a sharp projection (0); With a sharp projection (1).

72. Width of mid femur compared to width of tegmina in male: Narrower than tegmina width (0); Equal to tegmina width (1); Wider than tegmina width (2).

73. Margins of femora of three pairs of leg: All with a row of sharp teeth (0); Not with a row of sharp teeth (1).

74. Outer side of hind femur: With tubercles (0); Without tubercles (1).

75. Upper margin of hind femur before knee: With a large tooth (0); Without a large tooth (1).

76. Lower margin of hind femur: Smooth (0); Wavy (1); With minute serrations (2); With large tooth-like projections (3).

77. Base of mid tibia: Not enlarged (0); Enlarged (1).

78. Spines on hind tibia: Present (0); Absent (1).

79. Apical spurs of hind tibia: Lamellate (0); Spiniform (1).

80. Number of posterior tarsi: 1 segment (0); 3 segments (1).

81. Hind tibia and posterior tarsi: Not widened (0); Widened into a paddle-like shape (1).

82. Third segment of posterior tarsi in female: Normal, not enlarged (0); Ovate, enlarged (1)

83. Comparison of the length of the first and third segment of posterior tarsi: longer than (0); equal (1); shorter than (2).

84. Three pulvilli of first segment of posterior tarsi: Increase in turn (0); Equal in length (1).

85. The size of the three pulvilli of first segment of posterior tarsi: Normal (0); Extremely reduced, very small (1).

86. paraproctal lobe: 1 pair present (0); Absent (1).

87. Uncus of abdominal terminus in male: Present (0); Absent (1).

88. Posterior margin of female subgenital plate: With a triangular projection (0); With 3 teeth (1); Straight (2); Concave in the middle (3).

89. Dorsal margins of upper valvulae and ventral margins of lower valvulae: With fine serrations (0); Without fine serrations (1).

90. Hind tibia color: Brown (0); Black or black with pale rings (1)

91. Color of pronotal median carina and external lateral carinae: Without yellow or red spots (0); With yellow or red spots (1).

The matrix used in this study was:

26 93

Saussurella_cornuta 10031141?2000000200000111101300O-11110110010-
0100011103000200011001000011111200110011001110 00

Saussurella_longiptera 10031141?20000002000101111013000-11111110010-
010001110300020001100100001111120011000100 11?010

Bolivaritettix_circocephalus 00021141000000001001001101003010-210--110010-
010001110000011001100110000211120011000000110000

Eucriotettix_amplifemurus 10021141011100001011001001013010-110--
110030101000110100001100110011110021112001100000011?000

Thoradonta_yunnana 01022141320000000010011001033110-110--
110020101000000100110100110011110021002001100000011?010

Edentatettix_leyeensis 01023141120100101000111201003000-210--110010-

011001110000001101122110100-11110011001100110110

Xistra_zhengi 0002114111101201000111011003010-210--110010-

0100011110000000001100110100211120011001100110000

Gibbotettix_parvipulvillus 12023141320001112010111001113210-100--110010-

011001011201100111122111300-100300110000101111010

Paragavialidium_curvispinum 23023141320001101010011001003010-210--

1100202010011111-1010100110011230001003001100000011?010

Paragavialidium_hainanensis 23023141320001102010011001003010-210--

1100202010111111-1010100110011230001003001100000011?010

Scelimena_melli 23022141100000101010011101003000-210--

110020201000111101000100110011110001101001110000011?011

Systolederus_spicupennis 10022141130110100010001111003000-110--110010-

010001110000000001100110000211120011001100112010

Macromotettixoides_maoershanensis 01022141020000002000111211013120-110--

110010-0110011100000000011122111100-11100011000000110010

Macromotettixoides_orthomargina 01023141120000001000111021013110-210--

110010-0110011100000000001122113300-10130011000000110010

Zhengitettix_transpicula 10021141131110200010001101003010-210--

1100200010001110000000001100110000111120011001100111000

Euparatettix_tridentatus 10021141101100002000001111003000-210--110001-

0100011100000000011001100000111200110000000111000

Exothotettix_guangxiensis 01021131120000000022101120023230-110--110001-

011001-1-3020000011111100002111200110000000110000

Tetrix_japonica 01021141000000000000001111003000-210--110001-

0100011100000000011001100002111200110000000110000

Zhengitettix_triangularis 00021141131110200010011101003010-210--

110030001000111000000000110011000011112001100100011?000

Xya_shandongensis 0134214112000232-000010101003------0--110000-01-00-11010-

-20--0000010000-11100000-----0002110

Xya_sichuanensis 0134214110000232-000000101003------0--110000-01-00-11010--

20--0000010000-11100000-----0002100

Tripetalocera_ferruginea 112010413200000010021210-10-3--00110--110010-011011-1120012-1011221123002100300110000000110010

Formosatettix_leigongshanensis 02022141120000002002111121023230-020--110001-011001-1-302000111122111000-11120011000100110000

Systolederus_anhuiensis 100221411311101010000111111003000-110--110001-010001110000000000110011000011112001100000000110010

Systolederus_hainanensis 10021141131110100000011111003000-210--110001-0100011100000000001100110000011120011000000110010

Systolederus_prominemarginis 10022141131110100010011111003000-110--110001-01000111000000000011001100001111200110001000110010

Systolederus_spicupennis 100221411301101000100011111003000-110--110010-010001110000000000110011000021112001100110011201

Ergatettix_serrifemora 00022141001100000010011111013210-110--110001-01000111000101000110011001021102001100000111 0000
